# Social Determinants Influencing Nutrition Behaviors and Cardiometabolic Health in Indigenous Populations: A Scoping Review of the Literature

**DOI:** 10.3390/nu16162750

**Published:** 2024-08-17

**Authors:** Bishal Gyawali, George Frederick Mkoma, Stefanie Harsch

**Affiliations:** 1 Global Health Section, Department of Public Health, University of Copenhagen, 1014 Copenhagen, Denmark; 2 Department of Epidemiology Research, Statens Serum Institut, 2300 Copenhagen, Denmark; gefm@ssi.dk; 3 Section of Health Services Research, Department of Public Health, University of Copenhagen, 1353 Copenhagen, Denmark; 4 Center for Medicine and Society, Albert-Ludwigs-Universität Freiburg, 79098 Freiburg, Germany; stefanie.harsch@zmg.uni-freiburg.de

**Keywords:** social determinants of health, sociohistorical factors, nutrition behaviors, cardiometabolic health, indigenous populations

## Abstract

Nutrition behavior is influenced by a large number of factors, including social and cultural factors. This scoping review aims to summarize how social determinants of health (SDoH) influence nutrition behaviors in Indigenous populations affected by or at risk of cardiometabolic diseases. Following the PRISMA-ScR guidelines, we conducted a systematic search in six databases—PubMed, Web of Science, CINAHL, PsycINFO, Cochrane Library, and World Health Organization Global Index Medicus—limiting results to studies published in English up to 27 October 2023. A descriptive synthesis was conducted. We identified 1490 articles, and after screening, 31 of them met our inclusion criteria. We found that nutritional behavior is impacted by various SDoH domains, including economic stability, neighborhood and built environment, education, health and healthcare, and social and community context. The shift from traditional diets to Westernized diets and from subsistence-based food gathering to reliance on store-bought and processed foods reflects changes in SDoH, affecting both nutrition behaviors and health outcomes. Although not all included studies examined every SDoH domain in our review, future research should consider all domains to gain a comprehensive understanding of how they impact nutritional behavior. This approach will better inform interventions and policies, ultimately promoting health equity in Indigenous communities.

## 1. Introduction

Cardiometabolic diseases (CMDs), such as cardiovascular disease and type 2 diabetes, represent significant global health challenges in the 21st century [1]. These diseases disproportionately impact specific groups, including ethnic minorities, low-income individuals, and residents of economically disadvantaged areas [2]. Indigenous populations, characterized by their distinct cultural identities and strong ties to ancestral territories, are particularly vulnerable to health disparities in cardiometabolic health [3,4]. Compared to the general population, Indigenous people experience a higher burden of CMDs [5]. Poor nutrition greatly contributes to CMDs among Indigenous people. Improving nutrition quality can reduce these conditions by up to 50% [6].

Recent studies indicate that changes in nutritional behaviors have increased the burden of CMDs, with poor nutrition quality contributing to over 11 million deaths globally in 2017 [7]. Indigenous populations worldwide are undergoing a nutrition transition [8], characterized by a decline in traditional food consumption (foods native to the local environment) and a rise in the intake of market foods, including energy-dense and nutrient-poor products [9]. Research increasingly highlights that socioeconomic, cultural, and environmental factor—collectively known as social determinants of health (SDoH)—are crucial for understanding health disparities and promoting CMD prevention and management [10,11,12,13,14,15]. Since the seminal study on SDoH by Marmot et al., subsequent research has emphasized their significant role in influencing lifestyle, risk factors, and disease outcomes [16]. These determinants greatly influence nutritional habits, which are shaped by systemic factors, such as racism and unequal resource distribution [17]. Certain population subgroups, especially those who are vulnerable, marginalized, and disadvantaged, often live and work in more deteriorated environments. They face greater exposure to disease risk factors and experience physiological effects from chronic stress, leading to poorer health outcomes and shorter lifespans. Powell-Wiley et al. identified several dimensions of social determinants of health in marginalized populations, including economic stability, neighborhood and built environment, education access and quality, healthcare access and quality, and social and community context [11]. These determinants interact with cultural norms and traditions to shape eating habits [18]. 

Studying Indigenous populations presents challenges due to their diversity; the United Nations recognizes over 476 million Indigenous people across more than 5000 distinct groups globally [19]. Living conditions, historical injustices, colonization, land dispossession, and restricted access to traditional foods have significantly impacted Indigenous populations, influencing nutrition behaviors [20]. 

Considering the significant influence of SDoH on nutritional behavior and the gap in synthesizing these determinants within Indigenous populations, this scoping review aims to deepen our understanding of the social determinants of nutrition behavior among Indigenous populations living with or at risk of CMDs by summarizing existing evidence. The specific research question guiding this review was the following: ‘What are the social determinants of nutrition behavior among Indigenous populations living with or at risk of CMDs?’ Understanding these determinants is crucial for designing tailored interventions and comprehensive strategies to improve nutritional behavior in Indigenous populations.

## 2. Materials and Methods

This study employed the scoping review methodology, adhering to Arksey and O’Malley’s five-step process [21]. The methods are summarized according to the Preferred Reporting Items for Systematic Reviews and Meta-Analyses guidelines: Extension for Scoping Reviews (PRISMA-ScR) [22]. Following Arksey and O’Malley’s framework, the review steps included the following: (1) defining the research question; (2) identifying relevant studies; (3) selecting studies; (4) charting the data; and (5) collating, summarizing, and reporting the findings [21]. The PCC model (Population, Concepts, and Context) was used to construct the research question and develop the search strategy, following the Johanna Briggs Institute’s recommendations [23].

### 2.1. Identifying Relevant Studies

To identify relevant studies, a comprehensive search of six databases—PubMed, Web of Science, CINAHL, PsycINFO, Cochrane Library, and World Health Organization Global Index Medicus—was conducted. The search strategy employed medical subject headings and specific keywords (Supplementary File S1). Gray literature was also searched using identical terms in Google and Google Scholar. Identified publications were managed using EndNote 20 and screened for duplicates using Covidence (www.covidence.org). The search was limited to studies published in English from the inception of each database up to 27 October 2023. The search strategy was developed through consultation with a university librarian and discussions among the research team. 

### 2.2. Eligibility Criteria and Study Selection

For study selection, inclusion criteria focused on studies that (1) exclusively involved Indigenous populations; (2) discussed CMDs; (3) examined SDoH domains, including the economic, educational, neighborhood, health, and social factors influencing nutrition behavior (Supplementary File S2); and (4) conducted primary (original) research. Studies were excluded if they compared Indigenous with non-Indigenous groups, lumped different populations together under the term ‘Indigenous’ (e.g., studies describing nutritional transitions in a country), or focused solely on so-called tribal populations specifically named as such, or targeted local populations (e.g., a tribe in India or Kenya) that do not (self-)identify as Indigenous. Additionally, studies were excluded if they centered solely on the gut microbiome or other biomedical markers, reported CMD prevalence without linking it to nutrition, were not yet implemented (e.g., study protocols), or were review papers, opinion pieces, or intervention studies (Table S1). Search execution and screening were managed by one author (SH), with independent screening performed by two authors (SH and BG) who independently screened all articles using Covidence. Any discrepancies in article inclusion were resolved through consensus, with a third researcher (GFM) acting as a referee where necessary.

### 2.3. Charting the Data

The eligible articles were thoroughly reviewed multiple times by two researchers (BG and SH) to ensure familiarity with their content. Consensus was reached among researchers on the categories for data extraction. BG and SH extracted data from selected articles and then entered them into an Excel spreadsheet. The spreadsheet included the following categories: author(s), year of publication, study title, country, Indigenous group, general population or patients, study objectives, sample size and description, social determinants, nutritional behavior, CMD and/or CMD risk factors, and main findings.

### 2.4. Collating, Summarizing, and Reporting Results

A comprehensive analysis of the selected articles was conducted using the extracted data. A tabular summary of study details and outcomes was compiled. The extracted data were descriptively synthesized, providing an overview of the included study characteristics, settings, target group, and social determinants of nutritional behaviors. BG verified data accuracy. Quality appraisal of the included studies or meta-analysis was not undertaken, as this review aimed to provide an overview or mapping of relevant evidence on nutritional behavior and SDoH [23]. 

## 3. Results

### 3.1. Literature Search

**Figure 1:**
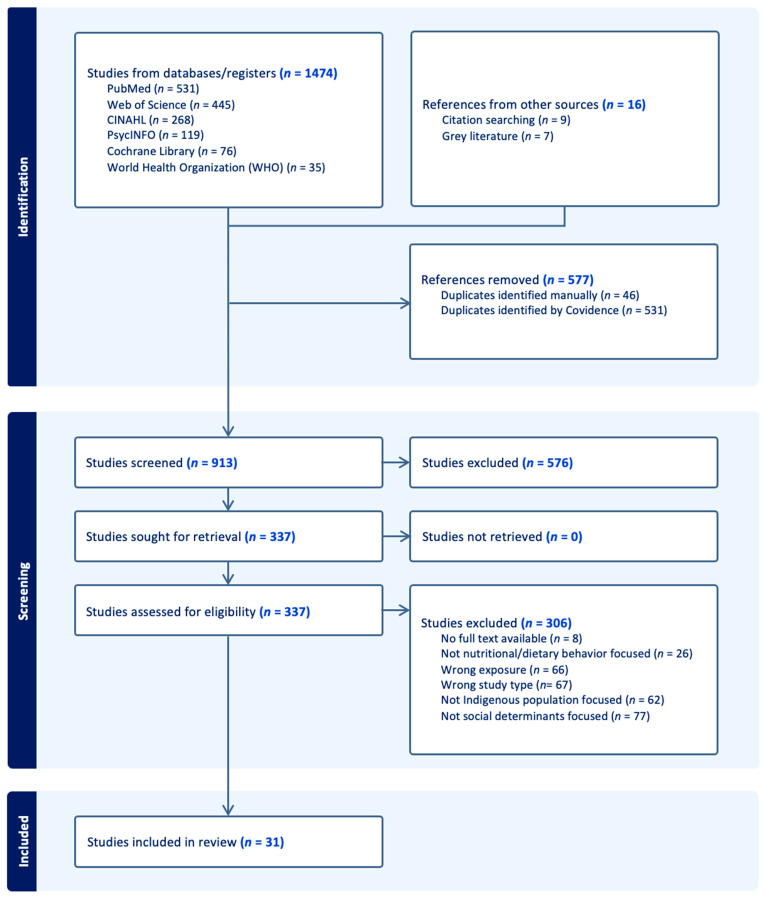
PRISMA study flow chart.

A total of 1474 articles were identified, primarily in the six electronic databases and an additional 16 articles from the reference check that met our study criteria. Moreover, 576 duplicate articles were removed, and the remaining 913 were subjected to screening according to the inclusion criteria. Following a review of the titles and abstracts, 337 were included for the subsequent full-text screening in accordance with the inclusion criteria. Finally, 31 articles met the inclusion criteria and were included in the current scoping review. Details of the screening process are illustrated in Figure 1.

### 3.2. Study Characteristics

**Figure 2:**
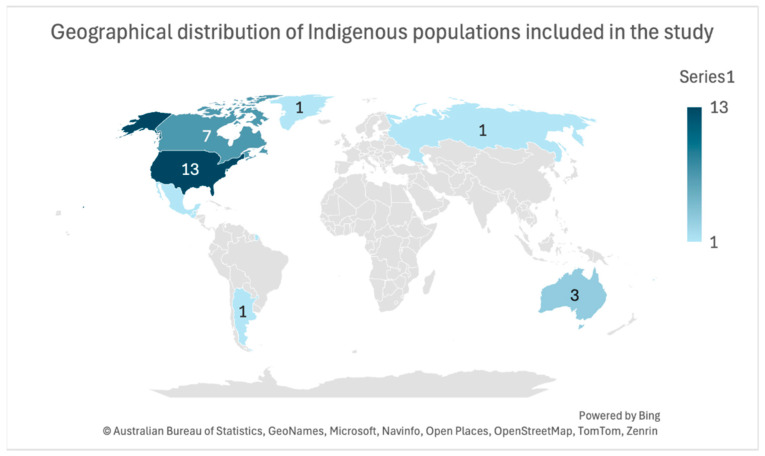
Geographical distribution of Indigenous populations included in this study. Please note that numbers for certain countries, such as French Guiana, Guatemala, Mexico, and Fiji, are not visible due to their small geographical size.

This review included peer-reviewed articles from ten countries focusing on various Indigenous populations. In the USA, the studies involved American Indian/Alaska Natives [24,25,26,27], American Indians from the Chickasaw and Choctaw Nations [28,29,30,31,32], Native Americans (Yup’ik) [33,34,35], and the Flathead Indian tribes [36]. Canadian studies covered First Nation [37,38], FN Anishina, Ojibwe, Aji-Cree [39], the Inuit from Nunavut [40,41], self-identified Indigenous populations [42], and the Woodland Cree [43]. Australian studies included isolated communities [44], Aboriginal groups [45], and Māori [14]. Additionally, this review included studies from Fiji (iTaukei) [46,47], Argentina (Toba and Wichí) [48], French Guiana (Palikur/Parikwene) [49], Greenland (Greenland Inuit) [50], Guatemala (Indigenous) [51], Mexico (Mayan) [52], and Russia (Yakutia) [53]. The geographical distribution of Indigenous populations included in the study is shown in Figure 2.

The publications ranged from 2005 to 2023, with the highest number in 2021 (seven articles). The study design included two mixed-methods studies, 16 quantitative studies, and 13 qualitative studies. The studies focused either on prevention for Indigenous communities at risk of CMD (22 studies) [14,27,28,29,30,33,34,35,36,37,38,39,41,42,43,44,46,47,48,49,50,53] or on disease management for people with CMD (9 studies) [24,25,26,31,32,40,45,51,52]. The characteristics of the included studies are detailed in Table 1, with full details in Table 2. 

### 3.3. Factors Influencing Nutrition Behaviors

#### 3.3.1. Sociohistorical Embedding of the Studies

Few included studies have examined how sociohistorical factors influence the social determinants of CMDs and their risk factors among Indigenous populations. These include nutritional changes prompted by diagnosed health conditions [34], shifts in dietary habits such as reduced consumption of traditional foods and increased intake of sugary beverages and non-nutritious foods [50], and lifestyle changes such as decreased traditional food gathering and increased adoption of Western diets [43]. Additionally, the contact with European societies in the 20th century accelerated these changes, leading to greater reliance on store-bought and processed foods [49]. The rapid expansion of the global food industry further altered food availability [52]. Historical factors such as trauma from colonization contribute to health disparities and poorer outcomes in nutrition-related chronic diseases [26]. Discrimination and limited access to culturally competent health services also impact health outcomes among populations [51], compounded by factors such as poverty and underfunded health programs [29].

#### 3.3.2. Dietary Characteristics

The included studies highlight diverse dietary patterns among Indigenous populations, which can be categorized by ecological and cultural distinctions. Geographical location plays a significant role in shaping traditional diets among Indigenous populations, which vary across distinct ecospheres: the cold, resource-limited north (Canada, Greenland, and Russia), hot temperature zones in Latin America, Fiji, and Australia, and semi-hot areas in the USA. Each climate zone dictates food availability; for instance, the northern regions traditionally include seal meat, arctic char, caribou meat, pasteurized milk, berries and spinach, often consumed fresh or preserved [40,41]. Woodland Cree communities in Canada also incorporate land animals, fish, berries, fats, mixed foods, grains, fruits, vegetables, and dairy products into their diets [43], while Yakutia in Russia relies on a subsistence diet rich in meat and dairy [53].

Despite traditional fare, the studies report widespread consumption of nutritionally poor, industrially processed foods among Indigenous communities. These include potato chips, frozen pizzas, refined flour products, cookies, and sugary beverages [41]. Similarly, Greenland Inuit have been observed consuming unhealthy options such as candy, cakes, and fast food [50]. Up to 80% of some diets consist of imported food items, reflecting a blend of traditional and store-bought products rather than a distinct categorization [42].

Traditional Indigenous diets in warmer ecological zones such as Argentina feature hunted game, wild honey, fruits, low-maintenance horticultural products, and store-bought items such as eggs and cheese [44,48]. Indigenous Australians in remote areas incorporate discretionary foods and sugary drinks alongside some fruits and vegetables, often resulting in a diet that is low in dietary fiber but high in fats and sugars [45]. Similar shifts from traditional diets to market foods have been observed among Indigenous groups in French Guiana and Mexico, resulting in increased consumption of sweet and fatty items [49,52].

Traditional foods of American Indians in the USA include Sochan, bean bread, and southern Appalachian foodways [31]. However, many American Indians and Alaska Natives frequently include less healthy options in their diets, such as processed meats, flour products, baked goods, soft drinks, fried potatoes, and fast foods [24]. Some studies report cultural practices contributing to dietary habits, including sweet tea consumption, cooking with fatback, and eating fried foods, reflecting both traditional and contemporary influences [28,29]. 

#### 3.3.3. Nutritional Behavior Outcomes

The included studies reported different nutritional behavior outcomes, including the following: (1) marketed and foraged food consumption [48], (2) dietary intake quality [35,44], (3) food or dietary intake [33,37,39,43,53], (4) food choices [24,28,29,31,32,40,41,49], (5) dietary patterns [27,42,50], (6) nutrition transitions (overconsumption and unhealthy eating) [46], (7) dietary regimens [52], (8) dietary modifications [51], (9) caloric restriction [14], (10) healthy eating practices [25,26,47], (11) dietary quality [36], (12) household food environment [34], (13) fruit and vegetable intake [30], (14) traditional food consumption [38], and (15) bush and store-bought food intake [45].

#### 3.3.4. Overview of Dietary Assessment Methods Used in Quantitative Studies

In this review, various quantitative studies utilized different methods to assess dietary intake. The dietary assessment methods included the 24 h food recall method, which was reported by four studies [39,42,43,48]. Additionally, four studies employed the Food Frequency Questionnaire (FFQ) method [24,33,35,50]. One study used the Harvard Food Frequency Questionnaire [53].

#### 3.3.5. SDoH Influencing Nutrition Behaviors

The most studied SDoH influencing nutrition behaviors was economic stability (n = 16), followed by social and community context (n = 15), neighborhood and built environment (n = 10), education (n = 5), and health and healthcare (n = 4) (Tables S2 and S3).

##### SDoH Influencing Nutrition Behaviors for Indigenous Populations Potentially at Risk

Economic stability as a determinant of nutrition behaviors among the general population at risk of CMD was reported by five quantitative studies [37,42,44,48,53], four qualitative studies [29,33,41,46], and one mixed-methods study [43]. Education as a factor affecting nutrition behavior was mentioned in one qualitative study [29]. Neighborhood and built environment were explored in two quantitative studies [39,44], two qualitative studies [29,41], and one mixed-methods study [43]. Health and healthcare were discussed in one qualitative study [14] and one mixed-methods study [43]. Social and community context was reported by two quantitative studies [33,50], four qualitative studies [29,33,41,46], and one mixed-methods study [43].

Several specific findings were noted. Higher socioeconomic status was found to correlate with increased consumption of marketed foods, suggesting that those with greater financial resources are more likely to purchase commercially available food items [48]. Unemployment was linked to lower dietary intake quality, indicating that lack of employment negatively affects food choices and overall diet [44]. Lower household income was consistently associated with poorer dietary intake quality and unhealthy dietary practices, highlighting the financial barriers to maintaining a healthy diet [37,44]. Modern lifestyle linked with higher income was positively associated with market and mixed diets, showing that increased income facilitates the adoption of diverse dietary patterns [53]. Shifts from traditional hunting-based economies to wage-based employment significantly impacted food availability and dietary choices, leading to reduced access to traditional, nutritious food [41]. Individuals in lower socioeconomic groups faced restricted access to nutritious foods, often relying on cheaper, processed options due to financial constraints [29,46]. Additionally, transportation challenges and high food costs impeded the ability of these groups to make healthy food choices [29]. Proximity to stores and household crowding were also influential, with closer proximity to stores generally correlating with healthier diets, while limited access to healthy food options and difficulties in reaching stores posed significant barriers [29,39,41,44]. Human-enforced environmental changes, such as deforestation, disrupted traditional food sources and affected dietary patterns, further complicating the efforts to maintain healthy eating habits [43]. A lack of nutrition knowledge was linked to unhealthy food choices [29]. Cultural perceptions of biomedical healthcare also influenced dietary adherence, with historical factors complicating the acceptance of health advice [14]. Social factors, such as low social position, significantly influenced dietary patterns [50]. Societal pressures and cultural norms often promote food consumption as a symbol of love and affluence and a shared belief that food should not be wasted [29,46]. Finally, colonization and socio-cultural assimilation profoundly impacted food consumption choices, displacing traditional food practices that emphasized food sharing and social cohesion [41]. Detailed factors are also reported in Table S2.

##### SDoH Influencing Nutrition Behaviors for Indigenous Populations Living with CMDs

Five qualitative studies [25,26,31,32,51] and one mixed-methods study [52] identified economic stability, specifically poverty and cost of living, as factors influencing nutrition behaviors among patients with CMD. Education, both as formal education but also as informally acquired knowledge about a healthy diet, was reported as a factor by one quantitative study [35] and three qualitative studies [25,26,40]. Neighborhood and built environment were characterized by access to food, environmental conditions, and the missing availability of consumer information and were noted in one quantitative study [35] and four qualitative studies [25,26,31,51]. Health and healthcare in terms of dominating worldviews were discussed in three qualitative studies [26,40,45]. Seven qualitative studies [14,26,31,32,45,47,51] and one mixed-methods study reported social and community context as factors affecting nutrition behaviors [52]. 

Several notable findings were observed. Food insecurity and the high cost of healthy foods due to limited income emerged as significant barriers to adopting healthy eating practices [25]. The studies highlighted affordability issues associated with fresh, healthy foods and the higher costs of nutritious options, influencing dietary choices negatively across different communities [26,31,32,51]. Additionally, poverty-related food cultures were identified as barriers to maintaining consistent dietary regimens among patients [52]. Educational attainment was found to be positively correlated with better diet quality among patients, emphasizing the role of education in promoting healthier eating habits [35]. Limited cooking knowledge and inadequate health education services were identified as barriers to consuming fresh, healthy foods [25,26,40]. Specific gaps in knowledge regarding dietary choices, carbohydrate selections, and meal spacing were noted among patients [40]. Moreover, household food patterns were found to influence diet quality, with healthier household food environments correlating with improved dietary habits among patients [35]. Urban–rural disparities in access to healthful foods, such as fresh fruits and vegetables, were reported, along with challenges related to travel time and costs to markets, further complicating efforts to maintain a healthy diet [25,26,51]. Environmental conditions, including gardening challenges and lack of refrigeration, posed barriers to consuming fresh, healthy foods, illustrating the broader impact of environmental factors on dietary choices [25,26,51]. Societal and individual factors, such as cultural trauma and chronic diseases, were also identified as influencers of healthy eating behaviors, highlighting the complex interplay of social determinants on dietary practices [26,40,45]. Cultural beliefs and practices significantly influenced dietary management approaches and the acceptance of healthcare advice among patients, emphasizing the importance of cultural competency in healthcare settings [14,45,52]. Social and cultural factors played a crucial role in dietary adherence among patients, with community support systems and traditional food practices facilitating healthier eating habits [32,47,51]. Challenges related to balancing cultural dietary practices with health recommendations were noted, reflecting the need for culturally sensitive healthcare interventions [51]. Cultural beliefs regarding the origins of certain health conditions and the incorporation of high-calorie foods in traditional ceremonies were identified as specific barriers to dietary adherence, highlighting the complexity of integrating cultural practices with modern health advice [52]. For further details, please refer to Table S3.

## 4. Discussion

### 4.1. Summary of Findings

This scoping review is among the first to map evidence of SDoH affecting nutrition behavior and cardiometabolic health among Indigenous populations. Our review reported various transitioning processes, such as from pastoral to more urbanized lifestyles, from traditional to Westernized diets, and from subsistence-based food gathering to reliance on store-bought and processed foods. These transitions reflect changes in economic situations, cultural practices, and access to resources, all of which significantly influence nutrition behaviors and health outcomes. Similarly, factors such as economic stability, education, neighborhood and built environment, health and health care, and social and community contexts significantly influence nutrition behavior among Indigenous populations at risk and those with CMDs, with multiple studies having identified various overlapping factors.

**Figure 3:**
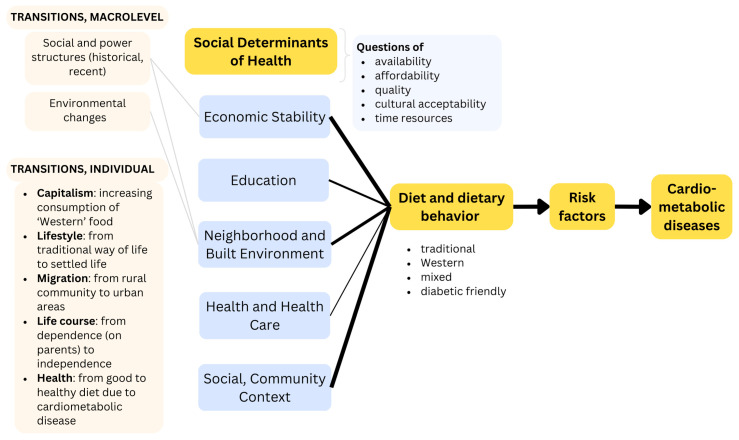
Concretizing the CMD causation pathway based on the empirical evidence found in the studies on Indigenous populations.

Figure 3 illustrates the SDoH-CMD causation pathways suggested by Powell-Wiley et al. [11] and complements it with the findings derived from the studies on Indigenous populations. The SDoH-related topics that emerged addressed questions regarding the availability and affordability of food, its nutritious value and quality, and its cultural acceptability. The thickness of the line corresponds to the proportion of studies reporting on the influence of this SDoH on diet and dietary behavior. Some included studies even provide explanations for this influence of SDoH on nutritional behavior, including social and power structures, as well as environmental changes. Furthermore, the studies report on five transitions linked to changes in the SDoH, including lifestyle, capitalism, migration, life course, and health changes.

### 4.2. Dietary Characteristics

Our review revealed that Indigenous populations exhibit diverse dietary characteristics, particularly traditional diets, which vary significantly across different ecospheres. Each ecosphere has its seasonality, which crucially affects the availability of traditional foods and consequently their contribution to dietary energy and intake estimation [54]. Importantly, our review underscores the benefits of traditional diets, including locally harvested animal and plant species, in maintaining a high-quality diet and promoting good health, as supported by other research [55]. However, our review also found a noticeable shift from traditional, nutrient-dense diets—high in fiber and low in fat and refined carbohydrates—to Westernized diets that are energy-dense and high in fat and refined sugars. The literature indicates that energy-dense, nutrient-poor foods are convenient and affordable, while healthy foods are often in limited supply and costly [56,57]. This shift has resulted in persistently poor dietary patterns and has significantly increased the prevalence of health conditions such as obesity, type 2 diabetes, and cardiovascular diseases, which were previously uncommon in Indigenous populations [9]. Our review underscores the vital role of traditional foods in Indigenous cultures, contributing to social, emotional, spiritual, and physical health [58]. Policy interventions developed with Indigenous people to increase access to traditional foods could help reduce these diet-related chronic diseases, such as CMDs and promote overall well-being.

### 4.3. SDoH Influencing Nutrition Behaviors for Indigenous Populations Potentially at Risk of CMDs

Our review revealed that economic factors such as low income, unemployment, and limited education are linked to a preference for cheaper, processed foods among the Indigenous populations at risk of CMDs. Research in Nunavut and Inuvialuit communities also supports this, showing that Inuit households with lower education and income levels, combined with limited access to nutrition education, consume fewer fruits and vegetables and rely more on energy-dense store-bought foods [59,60]. Addressing these economic disparities is crucial for improving dietary outcomes. The neighborhood and built environment also play significant roles in nutrition behavior. Proximity to stores affects access to fresh produce, with low-income neighborhoods often lacking access to fresh fruits and vegetables, preventing residents from meeting the recommended nutritional standards and highlighting a deficiency in local resources [61]. Colonization has had a profound impact on sociocultural assimilation and political activities, such as a ban on hunting, leading to a decline in traditional food practices among Indigenous populations. This decline negatively affects dietary habits. For instance, in the Nunavut community, restrictions on hunting caribou have limited access to sufficient traditional foods [62]. Malli et al. highlight how colonization has disrupted Indigenous food systems through capitalism, legal changes and sociocultural shifts [63]. The authors stress the importance of traditional knowledge sharing and authentic Indigenous inclusion in policymaking. Additionally, our review underscores the significant influence of cultural norms on dietary patterns. Research shows that Indigenous communities engage in food-sharing networks where surplus traditional foods are distributed to those in need, improving food access, especially in rural areas [64]. The erosion of these cultural practices may exacerbate mental health challenges and induce distress, both of which are risk factors for CMDs [65]. Empowering communities and adapting policies to ensure access to traditional foods are essential for promoting healthier dietary habits and fostering overall well-being in Indigenous populations and beyond.

### 4.4. SDoH Influencing Nutrition Behaviors for Indigenous Populations Living with CMDs

This scoping review found several SDoH that influence the nutrition behaviors of Indigenous people living with CMDs. The high cost of healthy foods, including fruits, vegetables, and diabetes-friendly products, was commonly reported as a barrier to healthy eating. Similar findings were reported in other studies, which indicated that these high costs led individuals to make food choices based on affordability rather than nutritional value, thereby impacting their ability to adhere to recommended diets [66]. Education also emerged as a crucial factor influencing nutrition behavior. In our review, higher educational levels were associated with better diet quality. Other literature confirms this finding, highlighting that the patients’ nutritional behavior improved with education on therapeutic diets, provided by clinicians [67]. Additionally, studies consistently reported that individuals with higher education levels understood nutritional information better than those with lower education levels [68,69]. Historical trauma from colonization also affected trust in healthcare providers, with mixed sentiments of distrust, skepticism, and respect influencing the willingness to follow dietary instructions. Similar findings were noted in other studies [70]. Urban–rural disparities in access to healthy foods were another barrier to nutrition behavior. Accessibility issues significantly influenced dietary patterns, with the rural areas facing greater challenges compared to the urban areas in our review. The studies included in our review further indicated that lack of transportation, long travel distances, and physical impairments hindered access to shopping centers and healthy foods, especially in rural areas. These findings were consistent with other studies, which highlighted physical barriers to healthy food among patients, compounded by poor health status and mobility impairments [66]. Contrary to other findings, one study in our review highlighted that urban dweller also faced barriers to healthy eating [25]. This was because urban-dwelling patients had less physical access and proximity to the lands where their traditional foods were fished, hunted, gathered, and grown. These neighborhoods and built environmental constraints call for innovative solutions, such as mobile food markets and community gardens, to improve food access among these groups of patients. Our review highlighted that cultural beliefs and social support significantly influenced dietary behaviors. Family members and caregivers played a vital role in promoting healthy eating habits, with traditional cuisine and home-farmed foods being often associated with good nutritional behavior. Similar findings were reported in other studies, which emphasized the strong influence of the family environment on food knowledge and preferences, particularly highlighting the roles of mothers and grandmothers [71]. Reduced social networks made participants particularly vulnerable to food insecurity and poor dietary intake. Additionally, evidence indicated that family involvement in meal preparation was associated with better disease management, including type 2 diabetes [72]. Reorienting health services to better consider Indigenous groups’ social organization and cultural values better could improve nutritional behavior and overall care. This might involve integrating family members into care models and developing approaches that align with the needs and preferences of these patient groups [73]. 

### 4.5. Limitations of This Review 

This review has several limitations. Firstly, relying solely on published literature is prone to publication bias and overlooks valuable unpublished studies. Additionally, restricting this review to English-language articles could exclude important research in other languages, limiting the inclusivity of the findings. The preponderance of cross-sectional studies in the reviewed literature could affect the depth and generalizability of the synthesized evidence. However, as this was a scoping review, our primary aim was to identify the existing evidence, describe its scope and extent, and highlight the gaps in the topic. There was also a lack of mechanistic studies that manipulated SDoH related to nutrition behaviors among Indigenous populations, resulting in their exclusion from our review. Furthermore, focusing on Indigenous populations means the findings cannot be generalized to the broader population at risk or living with CMDs, though it provides important empirical evidence relevant for Indigenous populations. Definitional challenges regarding the criteria for identifying Indigenous populations (see the UN decision on self-definition) [3] and the complexity of defining nutritional behavior [74] add further limitations. The heterogeneity in study designs and outcome measures across the included studies necessitates a cautious interpretation of the findings. Additionally, the individual studies included did not comprehensively address all domains of SDoH, and some studies did not explicitly explore their association with nutrition behaviors. This complexity hindered the precise identification of the SDoH factors influencing nutritional behaviors. This complexity has made it challenging to accurately identify and describe the exact strength of individual SDoH factors that influence nutritional behaviors. 

Despite these limitations, this review’s strength lies in its comprehensive synthesis of diverse studies from various databases, making it the first of its kind. This approach provides valuable insights into the SDoH affecting nutritional behavior among Indigenous populations. The rigorous screening and consultation processes enhance the validity and reliability of the findings, reinforcing the relevance of the conclusions and recommendations drawn from the included studies.

### 4.6. Implications and Recommendations for Future Studies

Our review highlights the significant relevance of social determinants—specifically economic stability, neighborhood and built environment, education, health and healthcare, and social and community context—in influencing the nutritional behavior and cardiometabolic health among Indigenous populations. The scope of our work enabled us to identify knowledge gaps in areas that are critical for developing targeted interventions to improve nutritional behavior in these communities. However, a cautious interpretation of our work is needed due to variations in methodologies, definitions of SDoH domains and Indigenous populations, as well as differences in the socioeconomic and cultural context of where the research was conducted.

Future research, including mechanistic studies, should thoroughly examine all SDoH domains to gain a comprehensive understanding and better inform interventions and policies, ultimately promoting health equity in Indigenous communities. Future quantitative and qualitative studies should clearly define outcome measures and report the direction of associations between the examined factors and nutritional behaviors, as this was lacking in the studies included in our review. Additionally, this review underscores the need for robust mixed-methods studies to gain a deeper understanding of the various SDoH pathways influencing dietary behaviors, particularly concerning cardiometabolic health in Indigenous populations, given that only two mixed-methods studies were found in our review. 

## 5. Conclusions

This scoping review summarizes comprehensive evidence to examine SDoH that influence nutritional behaviors in Indigenous populations affected by or at risk of cardiometabolic diseases. Nutritional behavior is impacted by various SDoH domains, including economic stability, neighborhood and built environment, education, health and healthcare, and social and community context. The shift from traditional diets to Westernized diets and from subsistence-based food gathering to reliance on store-bought and processed foods reflects changes in SDoH, affecting nutritional behaviors and health outcomes. Although not all included studies examined every SDoH domain, future research should consider all domains to gain a comprehensive understanding of how they concretely impact nutritional behavior and the interrelationships among different factors. This approach will better inform interventions and policies, ultimately promoting health equity in Indigenous communities.

## Figures and Tables

**Table 1:** Characteristics of included studies.

Sample Description		
Indicators	Data	Number of Studies
Country Indigenous group	USA	13
	American Indian/Alaska Natives	4
	American Indians (Chickasaw Nation, Choctaw Nation)	5
		3
	Native American (Yup’ik)	1
	Flathead Indian	1
	Canada	7
	First Nation	2
	FN Anishina, Ojibwe, Aji-Cree	1
	Indigenous Population (self-identified)	1
	Inuit (Nunavut Inuit)	2
	Woodland Cree	1
	Australia	3
	Isolated Australian Communities	1
	Aboriginal	1
	Māori	1
	Fiji; iTaukei	2
	Argentina: Toba and Wichí	1
	French Guiana: Palikur/Parikwene	1
	Greenland: Greenland Inuit	1
	Guatemala: Indigenous	1
	Mexico: Mayan	1
	Russia: Yakutia	1
Year	2005–2009	4
	2010–2014	3
	2017	2
	2018	2
	2019	5
	2020	4
	2021	7
	2022	1
	2023	3
Study Design	Mixed-methods study	2
	Quantitative study	16
	Qualitative study	13
Target Group	People with disease	9
	General population at risk	22

**Table 2:** Detailed characteristics of included studies.

Author	Study Year	Country	Indigenous Group	Study Population	Sample Size and Description	Study Design	Study Aim	Results/Findings Related to Nutrition Behavior
Indigenous populations potentially at risk of CMDs								
(Akande et al., 2021) [41]	2021	Canada	Inuit (Nunavut Inuit)	General population, possibly at risk	16 adults (10 women, six men)	Qualitative study involving semi-structured photo-elicitation interviews	To explore the perspectives of Nunavut Inuit on the barriers and enablers of healthy diets and physical activity participation in the community of Iqaluit	-Work-related changes, from hunting to a wage economy, influence food availability, impacting dietary choices. -Affordability is the main perceived barrier to healthy food choices, affecting traditional and non-traditional healthy foods. -Unhealthy junk foods are cheaper, while traditional foods have become more expensive due to the rising cost of hunting equipment and skilled hunters. -The availability of funds for purchasing healthy food is limited by spending choices such as smoking, drug use, and alcohol consumption. -The availability of healthy food options (including traditional foods) is a major barrier to eating healthily. -Political restriction on the number of specific wild animals allowed to be hunted reduces the consumption of healthy hunted meat. -Colonization and sociocultural assimilation have influenced food consumption practices, making former ‘food sharing’ practices less common.
(Bell et al., 2017) [14]	2017	Australia	Māori	General population, possibly at risk	15 Indigenous (Māori) people	Qualitative study involving narrative interviews	To identify the intrinsic mechanisms that specifically relate to Indigenous people’s interpretation of obesity	-Western models of calorie counting, diet and exercises were perceived as not sensitive to the needs and unrelatable concepts in the context of obesity. -The perceived association of biomedical health care with colonization causes feelings of alienation and reduces the acceptance of the health professional’s advice regarding a healthy diet.
(Berg et al., 2012) [28]	2012	United States of America	American Indians	General population, possibly at risk	998 American Indians	Quantitative study	To examine factors related to engaging in at least four days of physical activity per week and factors related to consuming at least five fruits and vegetables per day among a sample of American Indians in the Midwest	Education, knowledge, and perceptions are critical factors in improving nutrition behaviors.
(Bjerregaard and Larsen, 2021) [50]	2021	Greenland	Greenland Inuit	General population, possibly at risk	2436 Inuit aged 15+ years	Quantitative study	To explore the role of social position in dietary patterns and expenditures on food and other commodities	-Low social position associated with the selection of unhealthy food patterns.
(Bruner and Chad, 2014) [43]	2014	Canada	Woodland Cree	General population, possibly at risk	279 (females 15 years and older), 19 for interviews	Mixed-methods study	To explore the social, cultural, behavioral, and environmental factors influencing diet intake from a trans-generational perspective and to characterize the dietary practices among Woodland Cree women	-Shifts in the consumption of food associated with their Indian culture and an increase in ‘store-bought’ fast foods and overeating contributed to unhealthy bodies. -Younger community members prefer store-bought foods which are less healthy than hunting. -High costs to travel a long distance (145 km) to purchase food in the closest marketplaces influence food choices (e.g., fresh food would be spoiled). -Availability of healthy food options is limited locally, leading to the high frequency of purchasing packaged processed foods. -Environmental changes (e.g., deforestation) negatively influence hunting and thus make obtaining traditional foods more difficult. -A health center routinely supplies fruits and vegetables to individuals/programs, which is well received, yet this is not often possible due to long-distance traveling (300 km) to acquire these items. -Shifts in the consumption of food associated with their Indian culture and an increase in ‘store-bought’ fast foods and overeating contributed to unhealthy bodies. -Younger community members prefer store-bought foods which are less healthy than hunting.
(Buksh et al., 2022) [46]	2022	Pacific Island countries, Fiji	iTaukei mothers	General population, possibly at risk	15 Indigenous women	Qualitative study involving in-depth interviews	To explore sociocultural factors that contribute to overeating and unhealthy eating behaviors in an urban Indigenous community in Fiji	-Families with lower SES cannot afford meat and opt for cheaper processed meat options (canned meat, fish, sausages), thus eating less healthily. -Cultural norms, beliefs, expectations, and pressures contribute to overeating, unhealthy eating, and nutrition transitions among Indigenous populations in urban areas.
(Buksh et al., 2023) [47]	2023	Pacific Island countries, Fiji	iTaukei mothers (urban Indigenous Fijian mothers)	General population, possibly at risk	15 Indigenous women	Qualitative study involving in-depth interviews	To explore how urban indigenous Fijian mothers perceive healthy eating and how these perceptions impact the food decisions they make for their families	Multifaceted perceptions on healthy eating positively and negatively impacted the family food choices.
(Byker Shanks et al., 2020) [36]	2020	United States of America	Flathead Indian	General population, possibly at risk	Surveyed 79 residents and conducted 76 semi-structured interviews	Quantitative and qualitative multi- methods study	To document food environment experiences among residents of the Flathead Reservation in rural Montana	Perceptions of the food environment were linked to strategies that could be targeted to improve dietary quality.
(Domingo et al., 2021) [37]	2021	Canada	First Nations communities	General population, possibly at risk	3681 (2370 women/1311 men) First Nations people aged >= 19 years	Quantitative study	To examine the pattern of household food insecurity in First Nations communities and its association with obesity	Low income is linked to changes in unhealthy dietary practices. Receiving income support linked to healthy dietary practices.
(Estradé et al., 2021) [35]	2021	United States of America	Native American	General population, possibly at risk	580 tribal members, self-identified as the main household food purchaser (74% female)	Quantitative study	To identify psychosocial and household environmental factors related to diet quality among Native Americans (NA)	-Healthier household-level food patterns associated with higher diet quality. -High educational level associated with higher diet quality.
(Ho et al., 2008) [39]	2008	Canada	First Nations (Anishinaabe (Ojibwe and Oji-Cree)	General population, possibly at risk	129 First Nations adults	Descriptive quantitative study	To describe determinants of diet-related behavior and physical activity in First Nations for the development of culturally appropriate diabetes prevention programs	Larger households in remote communities tend to have higher scores for acquiring healthy food.
(Jock et al., 2020) [34]	2020	USA (Midwest, Southwest)	Native American	General population, possibly at risk	300 adults, three NA communities	Quantitative study	To describe the subgroups and demographic characteristics related to NA household food environments	There was low fruit and vegetable access among both the higher and lower access household food environments. Wild or brown rice and game meats were frequently obtained from higher access groups.
(Keith et al., 2018) [29]	2018	United States of America	American Indians	General population, possibly at risk	20 participants who were newly enrolled, academically underprepared tribal college students enrolled in life skills course	A nonexperimental cohort design used for qualitative descriptive analysis	To build an understanding of factors that influence healthy food choices among tribal college students at increased risk for college attrition	-Lack of income as students limit the acquisition of healthy foods. -Transportation challenges and high food costs are linked to difficulties in making healthy food choices. -Lack of nutrition knowledge linked with unhealthy food choices. -Difficulty accessing the store influences the likeliness to buy healthy foods. -Lack of exposure and positive role models for food choices. -A busy schedule is a barrier to preparing healthy meals at home. -Cultural traditions and practices are linked with healthy eating choices.
(Keshavarz et al., 2023) [42]	2023	Canada	Self-identified Indigenous people	General population, possibly at risk	1528 individuals in 2004 and 950 individuals in 2015	Quantitative study	To identify the dietary patterns of off-reserve Indigenous Peoples in Canada and their association with chronic conditions	High income positively correlated with higher adherence to healthy dietary patterns.
(Love et al., 2019) [30]	2019	United States, Oklahoma	American Indian Communities, Chickasaw Nation and the Choctaw Nation	General population, possibly at risk	513 American Indians	Quantitative study	To examine the relations between the perceived food environment, utilization of food retailers, fruit and vegetable intake, and chronic diseases, including obesity, hypertension, and type 2 diabetes among AI adults	57% of participants reported that it was easy to purchase fruits and vegetables in their town, and fewer (35%) reported that the available fruits and vegetables were of high quality. Additionally, over half (56%) reported traveling ≥20 miles round trip to shop for food.
(Philip et al., 2017) [33]	2017	United States, Alaska	Native population (Alaska) (Yup’ik)	General population, possibly at risk	486 Yup’ik adults	Quantitative study	To assess the relationships between socioeconomic, behavioral, and cardiometabolic risk factors among Yup’ik people of southwestern Alaska, with a focus on the role of the socioeconomic and cultural components	-Access to enough and appropriate foods is linked with better dietary practices. -Western culture is associated with higher consumption of processed foods and lower consumption of subsistence foods. -Western culture was associated with higher consumption of processed foods and lower consumption of subsistence foods.
(Rapinski et al., 2023) [49]	2023	French Guiana	Palikur/Parikwene People	General population, possibly at risk	75 community members, elders, healthcare professionals, administrators	Qualitative study, including ethnographic research and interviews	To identify the dietary patterns of off-reserve Indigenous men, women, and children in Canada and their association with chronic conditions in 2004 and 2015 while considering related sociodemographic and socioeconomic conditions	The income level among adults was recognized as an important factor that may be associated with the dietary intake of the off-reserve Indigenous population.
(Rosella et al., 2020) [38]	2020	Canada, Ontario	First Nations communities	General population, possibly at risk	993 adults	Cohort study	To predict 10-year diabetes risk and describe the factors that contribute to diabetes risk in First Nations adults living in Ontario First Nations communities	Factors included food insecurity, low income, and eating traditional vegetative foods.
(Setiono et al., 2019) [27]	2019	United States of America	American Indian Communities	General population, possibly at risk	580 adults from each of the six communities	Descriptive quantitative study	To characterize common dietary patterns among adults from 6 AI communities (N = 580) and assess their relationship with BMI, percentage body fat, waist-to-hip ratio, hypertension, and self-reported T2DM and cardiovascular disease	Five main dietary patterns: meat and fried foods, processed foods, fruits and vegetables, sugary snacks, and meat alternatives and high-protein foods. Those consuming more meat and fried foods had higher waist-to-hip ratios (0.03; 95% CI: 0.01, 0.04), BMI (2.45 kg/m2; 95% CI: 0.83, 4.07), and odds of being overweight or obese (OR: 2.63; 95% CI: 1.10, 6.31). Higher intake of processed foods was associated with increased odds of self-reported T2DM (OR: 3.41; 95% CI: 1.31, 8.90).
(Sorensen et al., 2005) [53]	2005	Russia, Northeastern Siberia	Yakutia	General population, possibly at risk	201 people in three urbanized towns and three rural communities	Descriptive quantitative study	To investigate diet and lifestyle determinants of plasma lipids in the Yakut, an Indigenous Siberian herding population	Modern lifestyles (often associated with higher incomes) correlated positively with market and mixed diets, while subsistence lifestyles (often associated with lower incomes) negatively correlated with market diets but positively correlated with mixed and subsistence diets.
(Stotz et al., 2021a) [25]	2021	United States of America	American Indian, Alaska Natives	General population, possibly at risk, possibly at risk	29 AI/AN with T2DM, 22 family members, 10 community-based key informants	Qualitative study involving focus groups and key informants’ interviews	To examine stakeholder perspectives on food insecurity and associated challenges to healthy eating among American Indian and Alaska Native Adults with T2DM	-Food insecurity was reported as a barrier to healthy eating practices. -High cost of healthy food and limited income linked with unhealthy food choices. -Living in rural areas is linked to a lack of access to healthful foods such as fruits and vegetables, supermarkets, and full-scale grocery stores, and to the higher availability of fast and processed foods. -Lack of fresh fruits and vegetables at grocery stores and non-availability of traditional foods and food-acquisition habits are barriers to healthy eating. -Strong community and family support systems, traditional foods, and food acquisition and preparation practices facilitate healthy eating.
(Stotz et al., 2021b) [26]	2021	United States of America	American Indian, Alaska Native Adults	General population, possibly at risk, possibly at risk	Nine experts in diabetes education, 20 community-based key informants, 29 AI/AN and 22 family members	Qualitative study involving key-informant interviews and focus groups	To understand stakeholder perspectives on facilitators and barriers to healthy eating for AI/AN adults with T2D to inform the cultural adaptation of an existing diabetes nutrition education curriculum	-Low cost associated with barriers to consuming fresh healthy food Urban dwellers experience barriers to healthy eating compared to rural dwellers. -Challenges with gardening are associated with barriers to consuming fresh healthy food. -Both individual factors (e.g., comorbidities and chronic diseases) and societal factors (e.g., trauma related to colonization) influence the ability to eat healthfully.
(Valeggia et al., 2010) [48]	2010	Argentina	Two Indigenous populations of the Argentine Gran Chaco: the Toba and Wichı	General population, possibly at risk	541 adults	Quantitative study	To evaluate the association between socioeconomic and nutritional statuses in adults of two Indigenous populations of the Argentine Gran Chaco: the Toba and Wichı’ of the province of Formosa	-Higher socioeconomic status linked to high consumption of marketed foods.
(Wycherley et al., 2019) [44]	2019	Australia	Indigenous Australians living in remote areas	General population, possibly at risk	13 remote Indigenous Australian communities, with populations ranging from 139–1079 persons	Quantitative study	To explore the modifiable environmental-level factors associated with the features of dietary intake that underpin cardiometabolic disease risk in this population group	-Unemployment linked to lower dietary intake quality. -Lower household income is associated with poorer dietary intake quality. -Lesser household crowding and shorter distances to neighboring stores are associated with lower dietary intake quality.
Indigenous populations living with CMDs								
(Bird et al., 2008) [40]	2008	Canada	Inuit	Adults with T2DM	Four ethnographic and informal interviews	Qualitative multi-case study, including ethnographic research, as well as informal interviews and field observations	To explore the experience of adult members of a small Arctic community who are living with diabetes as well as factors that influence their food choices and perceptions of diabetes and health management	-Lack of education and uncertainty about the proper carbohydrate choices and meal spacing. -Adaptability of T2DM patients to respond to their health condition is increased by learning about coping strategies, including healthy eating, and sharing knowledge to improve healthy eating. -Mixed sentiments about experiences with the ‘Southern’ style of healthcare, e.g., distrust, skepticism, trust, and respect, which influence the following of the healthcare providers’ instruction on a healthy diet.
(Dussart, 2009) [45]	2009	Australia	Aboriginal	Adults with T2DM	84 Aboriginal diabetic sufferers, kin and medical staff	Qualitative semi-structured interviews	To better understand how diabetes sufferers cope with their illness in everyday life for creating more culturally sensitive health promotion initiatives	-Biomedical imperatives (about an appropriate diet for the management of diabetes) are clashing with Indigenous forms of sociality. -Due to social expectations of generosity and sharing food, store-bought prepared food relieves the stress. -Initiatives to introduce dietary changes must find a balance between personal autonomy and social obligations.
(Goins et al., 2020) [31]	2020	United States of America	American Indians	Adults with T2DM	28 participants, 57% women	Qualitative study using a low-inference descriptive design with semi-structured in-depth interviews	To examine the beliefs, attitudes, and practices of older American Indians regarding their T2DM management	-Higher costs of foods linked with unhealthy food choices. -Difficulty of grocery shopping in terms of reading labels linked to determining the best food choices. -T2DM management influenced by sociocultural factors, Native culture, southern Appalachian culture, spirituality, traditional Native foods, southern Appalachian foods and foodways; social aspects of food, historical trauma, and financial circumstances related to food.
(Juárez-Ramírez et al., 2019) [52]	2019	Mexico	Mayan people	Adults with T2DM	195 adults with T2DM	Mixed-methods study	To understand non-adherence to medically recommended diets among Mayans with diabetes	- Cultural beliefs and not nutrition explain the origin of diabetes; therefore the relevance of food is overlooked. -High-calorie foods (corn, pork, sugar-based foods) are part of traditional ceremonies and make it difficult to follow dietary regimens.
(Schure et al., 2019) [32]	2019	United States of America	American Indians	Adults with T2DM	28 noninstitutionalized older tribal members aged >60 years	Qualitative study involving semi-structured in-person interviews	To examine dietary-related beliefs and self-management among older American Indians with T2DM	-Cultural upbringing of not wasting food hinders diabetic patients from eating healthily. -Social support, motivation, community dinners, healthcare professional and family influence, and personal beliefs (e.g., distaste for wasting food) facilitate adherence to a healthy diet.
(Teufel-Shone et al., 2018) [24]	2018	United States of America	Several American Indian, Alaska Natives	Adults with T2DM	2484 AI/AN with T2DM	Quantitative study	To examine the association between food choice and distress in a large national sample of American Indians/Alaska Natives with T2DM	Both males (34.9%) and females (65.1%) had higher healthy food scores than unhealthy scores. Unhealthy food scores showed significant positive relationships with distress for both genders (females: *β* = 0.078, *p* = 0.0007; males: *β* = 0.139, *p* < 0.0001).
(Wilson et al., 2021) [51]	2021	Guatemala	Indigenous Guatemalan community	Adults with T2DM	32 adults with T2DM	Qualitative structured interviews	To assess barriers to making dietary modifications for people living with T2DM in a rural Indigenous Guatemalan population	-A healthful diet is too costly. -Fluctuation of income level in ‘off-season’ times affects money available for healthy food. -Travel time and travel costs to the next market (5 to 30 km away) limit a healthy diet. -Lack of refrigerators limits the amount of perishable, fresh food that can be bought at a distance. -Challenges exist in the necessity to prepare food differently for diabetic patients than family members (incompatibility with family and traditional diet).

## Data Availability

Not applicable.

## Supplementary Materials

The following supporting information can be downloaded at: https://www.mdpi.com/article/10.3390/nu16162750/s1, File S1: Example search strategy PubMed; File S2: Key themes of Social Determinants of Health; Table S1: Inclusion and exclusion criteria; Table S2: SDoH influencing nutrition behaviors for Indigenous population potentially at risk of CMDs [14,29,33,37,39,41,42,43,44,46,48,50,53,56]; Table S3: SDoH influencing nutrition behaviors for Indigenous people living with CMDs [14,25,26,31,32,35,40,45,46,51,52].
