# Supplementary material for: Social Determinants Influencing Nutrition Behaviors and Cardiometabolic Health in Indigenous Populations: A Scoping Review of the Literature
Source: Nutrients. 2024 Aug 17;16(16):2750. doi: 10.3390/nu16162750 (PMC11356862; doi:10.3390/nu16162750)
Supplement: Supplementary file 1 [file nutrients-16-02750-s001.zip › nutrients-3134806-Supplementary file 1.pdf]

Supplementary file 1
Example search strategy PubMed:

Search string for Indigenous populations

(aborigin*[Title/Abstract] OR American Indian[Title/Abstract]) AND American Indians[Title/Abstract] OR eskimo*[Title/Abstract] OR first nation [Title/Abstract] OR first nations [Title/Abstract] OR greenlandic [Title/Abstract] OR Indigenous[Title/Abstract] OR inuit*[Title/Abstract] OR inupiat*[Title/Abstract] OR inuvialuit*[Title/Abstract] OR kalaallit*[Title/Abstract] OR maori[Title/Abstract] OR maoris[Title/Abstract] OR mapuche*[Title/Abstract] OR native American [Title/Abstract] OR native Americans[Title/Abstract] OR native people[Title/Abstract] OR native populations[Title/Abstract] OR native population[Title/Abstract] OR native siberian[Title/Abstract] OR native Siberians[Title/Abstract] OR navaho*[Title/Abstract] OR nunangat*[Title/Abstract] OR sami*[Title/Abstract] OR skolt*[Title/Abstract] OR taiga*[Title/Abstract] OR torres strait islander[Title/Abstract] OR tribe[Title/Abstract] OR tribal[Title/Abstract] OR yuit[Title/Abstract] OR yupik[Title/Abstract] OR zuni[Title/Abstract])

Search string for cardiometabolic diseases

cardiovascular[Title/Abstract] OR diabetes[Title/Abstract] OR hypertension[Title/Abstract] OR cardiometabolic*[Title/Abstract] OR obesity[Title/Abstract] OR heart attack"[Title/Abstract] OR stroke[Title/Abstract] OR insulin resistance"[Title/Abstract]

Search string for nutrition behavior

nutrition[Title/Abstract] OR eating behavior[Title/Abstract] OR eating behaviour[Title/Abstract] OR diet[Title/Abstract] OR dietary patterns[Title/Abstract]

Search string for social determinants of health

social determinants of health[Title/Abstract] OR econom*[Title/Abstract] OR environment*[Title/Abstract] OR neighborho*[Title/Abstract] OR education[Title/Abstract] OR "healthcare access"[Title/Abstract] OR social[Title/Abstract] OR community[Title/Abstract] OR Socioeconomic status[Title/Abstract] OR SES[Title/Abstract] OR housing[Title/Abstract] OR violence[Title/Abstract] OR noise[Title/Abstract] OR "food insecurity"[Title/Abstract] OR "early childhood adversity"[Title/Abstract] OR "social isolation"[Title/Abstract] OR discrimination[Title/Abstract]
