# Supplementary material for: Social Determinants Influencing Nutrition Behaviors and Cardiometabolic Health in Indigenous Populations: A Scoping Review of the Literature
Source: Nutrients. 2024 Aug 17;16(16):2750. doi: 10.3390/nu16162750 (PMC11356862; doi:10.3390/nu16162750)
Supplement: Supplementary file 1 [file nutrients-16-02750-s001.zip › nutrients-3134806-Supplementary file 2.pdf]

Supplementary file 2
Key themes of Social Determinants of Health:

Economic stability pertains to employment opportunities, poverty, cost of living, access to resources, income inequality, and debt.

Education includes educational attainment, access to quality education, early childhood education, higher education enrollment, high school graduation rates, and vocational training.

Neighborhood and built environment involve physical and safety conditions, environmental quality, housing/worksite conditions, crime and violence, food access, recreational facilities, and transportation systems.

Health and healthcare address availability, quality, and affordability of healthcare, provider availability, care affordability, health literacy, cultural competency, and care quality.

Social and community context encompasses discrimination, incarceration, social cohesion, culture, and civic participation.
