# Supplementary material for: Social Determinants Influencing Nutrition Behaviors and Cardiometabolic Health in Indigenous Populations: A Scoping Review of the Literature
Source: Nutrients. 2024 Aug 17;16(16):2750. doi: 10.3390/nu16162750 (PMC11356862; doi:10.3390/nu16162750)
Supplement: Supplementary file 1 [file nutrients-16-02750-s001.zip › nutrients-3134806-Supplementary Table S1.pdf]

**Supplementary Table S1:**

**Inclusion and exclusion criteria**

| **Inclusion Criteria** | **Exclusion Criteria** |
|---|---|
| **Population** | |
| ● Targeting exclusively Indigenous groups<br>● Either defined as Indigenous people from others, such as the original inhabitants of New Zealand, Australia, Canada or the USA or self-defined as 'Indigenous' following the UN definition of 'Indigenous people' | ● Studies that include an Indigenous group compared to a non-Indigenous group<br>● Studies that compare different populations, where 'Indigenous' is one group (e.g., studies describing dietary transitions in a country)<br>● Tribal studies that focus on local populations (e.g. a tribe in India or Kenya) that do not self-define as Indigenous |
| **Concept** | |
| ● Studies embedded in the discourse on CMDs<br>● Studies assessing nutritional behavior, including food intake, diversity, dietary patterns, eating habits, preferences, choices, and feeding-related practices | ● Studies focusing (only) on the gut microbiome and other biomedical markers |
| **Context** | |
| ● Economic stability, education, neighborhood, health and social factors that affect nutrition behavior or dietary practices | |
| **Study Characteristics** | |
| ● Original studies that are quantitative, qualitative, or mixed- methods, either cross-sectional or longitudinal. | ● Studies reporting the prevalence of CMDs without linking them to nutrition<br>● Studies not yet implemented (e.g. study protocol)<br>● Other article types excluded:<br>○ Review studies<br>○ Opinion papers<br>○ Intervention studies |
