# Supplementary material for: Social Determinants Influencing Nutrition Behaviors and Cardiometabolic Health in Indigenous Populations: A Scoping Review of the Literature
Source: Nutrients. 2024 Aug 17;16(16):2750. doi: 10.3390/nu16162750 (PMC11356862; doi:10.3390/nu16162750)
Supplement: Supplementary file 1 [file nutrients-16-02750-s001.zip › nutrients-3134806-Supplementary Table S2.pdf]

Supplementary Table S2: SDoH influencing nutrition behaviors for Indigenous population potentially at risk of CMDs

Factors	QN	QL	MM	Findings
**Economic stability**	5			<u>Findings quantitative</u><br>**Socioeconomic status**<br>• Higher socioeconomic status linked to high consumption of marketed foods [48].<br>**Employment opportunities**<br>• Unemployment linked to lower dietary intake quality [44].<br>**Poverty**<br>• Lower household income associated with poorer dietary intake quality [44].<br>• Low income linked to changes in unhealthy dietary practices [37].<br>• High income positively correlated with higher adherence to healthy dietary patterns [42].<br><br>**Access to adequate resources**<br>• Receiving income support linked to healthy dietary practices [37].<br>**Income inequality**<br>• Modern lifestyle (often associated with higher income) correlated positively with market and mixed diets, while subsistence lifestyle (often associated with lower income) negatively correlated with market diet but positively correlated with mixed and subsistence diets [53].
		4		<u>Findings qualitative</u><br>**Employment opportunities**<br>• Work-related changes from hunting to a wage economy influence food availability, impacting dietary choices [41].<br>**Poverty**<br>• Families with lower SES cannot afford meat and opt for cheaper processed meat options (canned meat, fish, sausages) thus eat less healthily [46].<br>• Lack of income as students limits the acquisition of healthy foods [29].<br>• Affordability is the main perceived barrier to healthy food choices, affecting both traditional and non-traditional healthy foods [41].<br>• Unhealthy junk foods are cheaper, while traditional foods have become more expensive due to the rising cost of hunting equipment and skilled hunters [41].<br>**Cost of living and transportation**<br>• Transportation challenges and high food costs are linked to difficulties in making healthy food choices [29].

				**Access to adequate resources**<br>• Access to enough and appropriate foods is linked with better dietary practices [33].<br>• The availability of funds for purchasing healthy food is limited by spending choices such as smoking, drug use, and alcohol consumption [41].
			1	Findings mixed-methods study<br>**Cost of living and transportation**<br>• High costs to travel a long distance (145 km) to purchase food in the closest market places influence food choices (e.g. fresh food would be spoiled) [43].
**Education**	0			Findings quantitative
		1		Findings qualitative<br>• Lack of nutrition knowledge linked with unhealthy food choices [29].
**Neighborhood and Built Environment**	2			Findings quantitative<br>**Access to food**<br>• Lesser household crowding and shorter distance to neighboring stores are associated with lower dietary intake quality [44].<br>• Larger households in remote communities tend to have higher scores for acquiring healthy food [39].
		2		Findings qualitative<br>**Access to food**<br>• Availability of healthy food options (including traditional foods) is a main barrier to eating healthily [41].<br>• Difficulty getting to the store influences the likeliness to buy healthy foods [29].<br>**Environmental conditions**<br>• Political restriction on the number of specific wild animals allowed to be hunted reduce the consumption of healthy hunted meat [41].
			1	Findings mixed-methods study<br>**Access to food**<br>• Availability of healthy food options is limited locally leads to the high frequency of purchasing of packaged processed foods [43].<br>**Environmental conditions**<br>• Environmental changes (e.g. deforestation) negatively influence hunting and thus makes obtaining traditional foods more difficult [43].

**Health and health care**	0			Findings quantitative
		1		Findings qualitative<br>• The perceived association of biomedical health care with colonization causes feelings of alienation and reduces the acceptance of the health professional's advices regarding healthy diet [14].
				Findings mixed-methods study<br>• Health center routinely supplies fruits and vegetables to individuals/programs which is well received, yet not often possible due to long-distance traveling (300 km) to acquire these items [43].
**Social and community context**	2			Findings quantitative<br>**Culture**<br>• Low social position associated with the selection of unhealthy food patterns [50].<br>• Western culture is associated with higher consumption of processed foods and lower consumption of subsistence foods [33].
		4		Findings qualitative<br>**Culture**<br>• Cultural norms, beliefs, expectations, and pressures contribute to overeating, unhealthy eating, and nutrition transitions among Indigenous populations in urban areas [46].<br>• Cultural traditions and practices are linked with healthy eating choices [29].<br>• Western culture was associated with higher consumption of processed foods and lower consumption of subsistence foods [33].<br>**Lifestyle**<br>• Busy schedule is a barrier to preparing healthy meals at home [29].<br>**Social Cohesion**<br>• Lack of exposure and positive role models for food choices [29].<br>• Colonization and socio-cultural assimilation have influenced food consumption practices, making former 'food sharing' practices less common [41].

			1	Findings mixed-methods study<br>**Culture**<br>• Shifts in the consumption of food associated with their Indian culture and an increase in 'store-bought' fast foods and overeating contributed to unhealthy bodies [43].<br>• Younger community members prefer store-bought foods which are less healthy than hunting [43].

QL: Qualitative studies; QN: Quantitative studies; MM: Mixed-methods studies

References:

14. Bell, R.; Smith, C.; Hale, L.; Kira, G.; Tumilty, S. Understanding obesity in the context of an Indigenous population-A qualitative study. *Obes. Res. Clin. Pract.* **2017**, *11*, 558–566. https://doi.org/10.1016/j.orcp.2017.04.006.

29. Keith, J.F.; Stastny, S.; Brunt, A.; Agnew, W. Barriers and Strategies for Healthy Food Choices among American Indian Tribal College Students: A Qualitative Analysis. *J. Acad. Nutr. Diet.* **2018**, *118*, 1017–1026. https://doi.org/10.1016/j.jand.2017.08.003.

33. Philip, J.; Ryman, T.K.; Hopkins, S.E.; O'Brien, D.M.; Bersamin, A.; Pomeroy, J.; Thummel, K.E.; Austin, M.A.; Boyer, B.B.; Dombrowski, K. Bi-cultural dynamics for risk and protective factors for cardiometabolic health in an Alaska Native (Yup'ik) population. *PLoS ONE* **2017**, *12*, e0183451. https://doi.org/10.1371/journal.pone.0183451.

37. Domingo, A.; Spiegel, J.; Guhn, M.; Wittman, H.; Ing, A.; Sadik, T.; Fediuk, K.; Tikhonov, C.; Schwartz, H.; Chan, H.M.; et al. Predictors of household food insecurity and relationship with obesity in First Nations communities in British Columbia, Manitoba, Alberta and Ontario. *Public Health Nutr.* **2021**, *24*, 1021–1033. https://doi.org/10.1017/s1368980019004889.

39. Ho, L.; Gittelsohn, J.; Sharma, S.; Cao, X.; Treuth, M.; Rimal, R.; Ford, E.; Harris, S. Food-related behavior, physical activity, and dietary intake in First Nations—A population at high risk for diabetes. *Ethn. Health* **2008**, *13*, 335–349. https://doi.org/10.1080/13557850701882936.

41. Akande, V.O.; Fawehinmi, T.O.; Ruiter, R.A.C.; Kremers, S.P.J. Healthy Dietary Choices and Physical Activity Participation in the Canadian Arctic: Understanding Nunavut Inuit Perspectives on the Barriers and Enablers. *Int. J. Environ. Res. Public Health* **2021**, *18*, 940. https://doi.org/10.3390/ijerph18030940.

42. Keshavarz, P.; Lane, G.; Pahwa, P.; Lieffers, J.; Shafiee, M.; Finkas, K.; Desmarais, M.; Vatanparast, H. Dietary Patterns of Off-Reserve Indigenous Peoples in Canada and Their Association with Chronic Conditions. *Nutrients* **2023**, *15*, 1485.

43. Bruner, B.G.; Chad, K.E. Dietary practices and influences on diet intake among women in a Woodland Cree community. *J. Hum. Nutr. Diet.* **2014**, *27* (Suppl. 2), 220–229. https://doi.org/10.1111/jhn.12121.

44. Wycherley, T.P.; van der Pols, J.C.; Daniel, M.; Howard, N.J.; O'Dea, K.; Brimblecombe, J.K. Associations between Community Environmental-Level Factors and Diet Quality in Geographically Isolated Australian Communities. *Int. J. Environ. Res. Public Health* **2019**, *16*, 1943. https://doi.org/10.3390/ijerph16111943.

46. Buksh, S.M.; de Wit, J.B.F.; Hay, P. Sociocultural Influences Contribute to Overeating and Unhealthy Eating: Creating and Maintaining an Obesogenic Social Environment in Indigenous Communities in Urban Fiji. *Nutrients* **2022**, *14*, 2803. https://doi.org/10.3390/nu14142803.

48. Valeggia, C.R.; Burke, K.M.; Fernandez-Duque, E. Nutritional status and socioeconomic change among Toba and Wichí populations of the Argentinean Chaco. *Econ. Hum. Biol.* **2010**, *8*, 100–110. https://doi.org/10.1016/j.ehb.2009.11.001.

50. Bjerregaard, P.; Larsen, C.V.L. Social determinants of dietary patterns, food basket costs and expenditure on alcohol and tobacco amongst Greenland Inuit. *Public Health Nutr.* **2021**, *24*, 4975–4984. https://doi.org/10.1017/s1368980020005133.

53. Sorensen, M.V.; Snodgrass, J.J.; Leonard, W.R.; Tarskaia, A.; Ivanov, K.I.; Krivoshapkin, V.G.; Spitsyn, V.A. Health consequences of postsocialist transition: Dietary and lifestyle determinants of plasma lipids in Yakutia. *Am. J. Hum. Biol.* **2005**, *17*, 576–592. https://doi.org/10.1002/ajhb.20426.

56. Buksh, S.M.; de Wit, J.B.F.; Hay, P. Sociocultural Influences Contribute to Overeating and Unhealthy Eating: Creating and Maintaining an Obesogenic Social Environment in Indigenous Communities in Urban Fiji. *Nutrients* **2022**, *14*, 2803. https://doi.org/10.3390/nu14142803.
