# Supplementary material for: Social Determinants Influencing Nutrition Behaviors and Cardiometabolic Health in Indigenous Populations: A Scoping Review of the Literature
Source: Nutrients. 2024 Aug 17;16(16):2750. doi: 10.3390/nu16162750 (PMC11356862; doi:10.3390/nu16162750)
Supplement: Supplementary file 1 [file nutrients-16-02750-s001.zip › nutrients-3134806-Supplementary Table S3.pdf]

Supplementary Table S3: SDoH influencing nutrition behaviors for Indigenous people living with CMDs

Factors	QN	QL	MM	Findings
**Economic stability**	0			Findings quantitative
		5		Findings qualitative<br>**Poverty**<br>• Food insecurity was reported as a barrier to healthy eating practices [25].<br>• High cost of healthy food and limited income linked with unhealthy food choices [25].<br>**Cost of living**<br>• Low cost associated with barriers to consuming fresh healthy food [26].<br>• Higher costs of foods linked with unhealthy food choices [31].<br>• Cost and affordability of healthier foods were barriers to healthy food choices [32].<br>• Healthful diet is too costly [51].<br>• Fluctuation of income level in 'off-season' times affects money available for healthy food [51].
			1	Findings mixed-methods study<br>**Poverty**<br>Poverty-based food culture as a barrier to low adherence to a diet regimen [52].
**Education**	1			Findings quantitative<br>• High educational level associated with higher diet quality [35]
		3		Findings qualitative<br>• Limited cooking knowledge as barriers to consuming fresh healthy food [26].<br>• Health education services to promote healthy eating among people living with diabetes [26].<br>• Lack of education & uncertainty about the proper carbohydrate choices and meal spacing [40].<br>• Adaptability of patients to respond to their health condition is increased by learning about coping strategies including healthy eating, sharing knowledge to improve healthy eating and thus prevent diabetes [40].
**Neighborhood and Built Environment**	1			Findings quantitative<br>• Healthier household-level food pattern associated with higher diet quality [35].

		4		Findings qualitative<br>**Access to food**<br>• Urban dwellers experience barriers to healthy eating compared to rural dwellers [26].<br>• Living in rural areas is linked to a lack of access to healthful foods such as fruits and vegetables, supermarkets, and full-scale grocery stores, and to the higher availability of fast and processed foods [26].<br>• Lack of fresh fruits and vegetables at grocery stores, and non-availability of traditional foods and food-acquisition habits as barriers to healthy eating [25].<br>• Travel time and travel cost to the next market (5 to 30 km away) limit healthy diet [51].<br>**Environmental conditions**<br>• Challenges with gardening associated with barriers to consuming fresh healthy food [26].<br>**Preserving healthy food**<br>• Lack of refrigerators limits the amount of perishable, fresh food that can be bought at a distant market [51].<br>**Consumer information**<br>• Difficulty of grocery shopping in terms of reading labels linked to determining best food choices [51].
**Health and health care (accessibility and health literacy)**	0			Findings quantitative
		3		Findings qualitative<br>• Both individual factors (e.g., comorbidities and chronic diseases) and societal factors (e.g., trauma related to colonization) influence the ability to eat healthfully [26].<br>• Mixed sentiments about experiences with the 'Southern' style of healthcare, e.g. distrust, skepticism, trust, and respect, which influence the following of the healthcare providers' instruction on healthy diet [40].<br>• Biomedical imperatives (about an appropriate diet for management of diabetes) are clashing with Indigenous forms of sociality [45].
	0			Findings quantitative

**Social and community context (social cohesion, discrimination, incarceration)**		7		Findings qualitative<br>**Culture**<br>• Mothers are sociocultural agents setting eating-appropriate standards for families and possess the local knowledge that (most) traditional cuisine and own-farmed food is healthy and needs to be prioritized over unhealthy eating [46].<br>• Western models of calorie counting diet and exercises were perceived as not sensitive to the needs and unrelatable concepts in the context of obesity [14].<br>• Cultural upbringing of not wasting food hinders diabetic patients to eat healthily [32].<br>• Due to social expectation of generosity and sharing food, store-bought prepared food relieves the stress [45].<br>• Initiatives to introduce dietary changes must find a balance between personal autonomy and social obligations [45].<br>• T2DM management was influenced by sociocultural factors, Native culture, southern Appalachian culture, spirituality, traditional Native foods, southern Appalachian foods and foodways; social aspect of food historical trauma, financial circumstances related to food [31].<br>**Social Cohesion**<br>• Strong community and family support systems, traditional foods, and food acquisition and preparation practices facilitate healthy eating [25].<br>• Social support, motivation, community dinners, healthcare professional and family influence, and personal beliefs (e.g., distaste for wasting food) facilitate adherence to a healthy diet [32].<br>• Challenges exist in the necessity to prepare food differently for diabetic patient than family member (incompatibility with family and traditional diet) [51].
			1	Findings mixed-methods study<br>**Culture**<br>• The origin of diabetes is explained by cultural beliefs and not nutrition, therefore the relevance of food is overlooked [52].<br>• High-calorie foods (corn, pork, sugar-based foods) are part of traditional ceremonies and make it difficult to follow dietary regimens [52].

QL: Qualitative studies; QN: Quantitative studies; MM: Mixed-methods studies

References

14. Bell, R.; Smith, C.; Hale, L.; Kira, G.; Tumilty, S. Understanding obesity in the context of an Indigenous population-A qualitative study. *Obes. Res. Clin. Pract.* **2017**, *11*, 558–566. https://doi.org/10.1016/j.orcp.2017.04.006.

25. Stotz, S.; Brega, A.G.; Henderson, J.N.; Lockhart, S.; Moore, K. Food Insecurity and Associated Challenges to Healthy Eating Among American Indians and Alaska Natives With Type 2 Diabetes: Multiple Stakeholder Perspectives. *J. Aging Health* **2021**, *33*, 31s–39s. https://doi.org/10.1177/08982643211013232.

26. Stotz, S.A.; Brega, A.G.; Gonzales, K.; Hebert, L.E.; Moore, K.R. Facilitators and Barriers to Healthy Eating Among American Indian and Alaska Native Adults with Type 2 Diabetes: Stakeholder Perspectives. *Curr. Dev. Nutr.* **2021**, *5*, 22–31. https://doi.org/10.1093/cdn/nzaa114.

31. Goins, R.T.; Jones, J.; Schure, M.; Winchester, B.; Bradley, V. Type 2 diabetes management among older American Indians: Beliefs, attitudes, and practices. *Ethn. Health* **2020**, *25*, 1055–1071. https://doi.org/10.1080/13557858.2018.1493092.

32. Schure, M.; Goins, R.T.; Jones, J.; Winchester, B.; Bradley, V. Dietary Beliefs and Management of Older American Indians With Type 2 Diabetes. *J. Nutr. Educ. Behav.* **2019**, *51*, 826–833. https://doi.org/10.1016/j.jneb.2018.11.007.

35. Estradé, M.; Trude, A.C.B.; Pardilla, M.; Jock, B.W.I.; Swartz, J.; Gittelsohn, J. Sociodemographic and Psychosocial Factors Associated With Diet Quality in 6 Rural Native American Communities. *J. Nutr. Educ. Behav.* **2021**, *53*, 10–19. https://doi.org/10.1016/j.jneb.2020.05.001.

40. Bird, S.M.; Wiles, J.L.; Okalik, L.; Kilabuk, J.; Egeland, G.M. Living with diabetes on Baffin Island: Inuit storytellers share their experiences. *Can. J. Public Health* **2008**, *99*, 17–21. https://doi.org/10.1007/bf03403734.

45. Dussart, F. Diet, diabetes and relatedness in a central Australian Aboriginal settlement: Some qualitative recommendations to facilitate the creation of culturally sensitive health promotion initiatives. *Health Promot. J. Austr.* **2009**, *20*, 202–207. https://doi.org/10.1071/he09202.

46. Buksh, S.M.; de Wit, J.B.F.; Hay, P. Sociocultural Influences Contribute to Overeating and Unhealthy Eating: Creating and Maintaining an Obesogenic Social Environment in Indigenous Communities in Urban Fiji. *Nutrients* **2022**, *14*, 2803. https://doi.org/10.3390/nu14142803.

51. Wilson, J.; Svenson, J.; Duffy, S.; Schmidt, J. Barriers to dietary modifications for people living with type 2 diabetes in a rural indigenous Guatemalan community. *Healthc. Low-Resour. Settings* **2021**, *9*.

52. Juárez-Ramírez, C.; Théodore, F.L.; Villalobos, A.; Allen-Leigh, B.; Jiménez-Corona, A.; Nigenda, G.; Lewis, S. The importance of the cultural dimension of food in understanding the lack of adherence to diet regimens among Mayan people with diabetes. *Public Health Nutr.* **2019**, *22*, 3238–3249. https://doi.org/10.1017/s1368980019001940.
